# Refining the resolution of craniofacial dysmorphology in bipolar disorder as an index of brain dysmorphogenesis

**DOI:** 10.1016/j.psychres.2020.113243

**Published:** 2020-09

**Authors:** Stanislav Katina, Brendan D. Kelly, Mario A. Rojas, Federico M. Sukno, Aoibhinn McDermott, Robin J. Hennessy, Abbie Lane, Paul F. Whelan, Adrian W. Bowman, John L. Waddington

**Affiliations:** a School of Mathematics and Statistics, University of Glasgow, Glasgow, UK; b Institute of Mathematics and Statistics, Masaryk University, Brno, Czech Republic; c Centre of Experimental Medicine, Slovak Academy of Sciences, Bratislava, Slovakia; d St. John of God Hospital, Stillorgan, Co., Dublin, Ireland; e Department of Psychiatry, Trinity Centre for Health Sciences, Tallaght University Hospital, Dublin, Ireland; f Centre for Image Processing & Analysis, Dublin City University, Dublin, Ireland; g Molecular & Cellular Therapeutics, Royal College of Surgeons in Ireland, Dublin, Ireland; h School of Medicine and Medical Sciences, University College Dublin, Dublin, Ireland; i Jiangsu Key Laboratory of Translational Research & Therapy for Neuro-Psychiatric Disorders, College of Pharmaceutical Sciences, Soochow University, Suzhou, China

**Keywords:** Bipolar disorder, Neurodevelopment, Craniofacial dysmorphology, Brain dysmorphogenesis, Geometric morphometrics

## Abstract

As understanding of the genetics of bipolar disorder increases, controversy endures regarding whether the origins of this illness include early maldevelopment. Clarification would be facilitated by a ‘hard’ biological index of fetal developmental abnormality, among which craniofacial dysmorphology bears the closest embryological relationship to brain dysmorphogenesis. Therefore, 3D laser surface imaging was used to capture the facial surface of 21 patients with bipolar disorder and 45 control subjects; 21 patients with schizophrenia were also studied. Surface images were subjected to geometric morphometric analysis in *non-affine* space for more incisive resolution of subtle, localised dysmorphologies that might distinguish patients from controls. Complex and more biologically informative, non-linear changes distinguished bipolar patients from control subjects. On a background of minor dysmorphology of the upper face, maxilla, midface and periorbital regions, bipolar disorder was characterised primarily by the following dysmorphologies: (a) retrusion and shortening of the premaxilla, nose, philtrum, lips and mouth (the frontonasal prominences), with (b) some protrusion and widening of the mandible-chin. The topography of facial dysmorphology in bipolar disorder indicates disruption to early development in the frontonasal process and, on embryological grounds, cerebral dysmorphogenesis in the forebrain, most likely between the 10^th^ and 15^th^ week of fetal life.

## Highlights

• Facial dysmorphology was studied as an accessible index of brain dysmorphogenesis.
• Bipolar patients showed complex, non-linear changes relative to control subjects.
• Bipolar disorder was characterised by retrusion of the frontonasal prominences.
• The findings indicate dysmorphogenesis in bipolar disorder during early fetal life.

## 1 Introduction

Increased understanding of the genetics of bipolar disorder is revealing shared genetic risk for other illnesses that include the neurodevelopmental condition of attention deficit/hyperactivity disorder as well as schizophrenia (Bipolar Disorder and Schizophrenia Working Group of the Psychiatric Genomics Consortium, 2018; Brainstorm Consortium, 2018). While the neurodevelopmental model continues to hold ‘centre stage’ in relation to schizophrenia (Waddington et al., 2012; Weinberger, 2017), controversy endures regarding the extent to which bipolar disorder might also have developmental origins (Sanches et al., 2008; Demjaha et al., 2012; Parellada et al., 2017).

Clarification of this controversy would be facilitated by a ‘hard’ biological index of developmental abnormality. Anatomical dysmorphologies, both major congenital abnormalities (Waddington et al., 2008) and minor physical anomalies (Xu et al., 2011), indicate developmental disruption during early fetal life; however, they are heterogeneous, difficult to quantify, and their status in bipolar disorder is evolving (Akabaliev et al., 2014; Berecz et al., 2017). Craniofacial dysmorphologies bear the closest embryological relationship to brain dysmorphogenesis (DeMyer et al., 1964; Schneider et al., 2001; Marcucio et al., 2015), but even when analysed anthropometrically (Lane et al., 1997; Deutsch et al., 2015) they lack the topographical resolution that can only come from detailed quantification of dysmorphology of the whole facial surface in its intrinsic 3-dimensional (3D) space.

We and others have previously applied 3D laser surface imaging and geometric morphometrics to resolve and quantify craniofacial dysmorphology in psychotic illness (Buckley et al., 2005; Hennessy et al., 2007) and subsequently we reported preliminary evidence for craniofacial dysmorphology in bipolar disorder (Hennessy et al., 2010). Recently, we have applied more advanced geometric morphometric techniques to craniofacial dysmorphology in 22q11.2 deletion syndrome, which carries an approximately 25-fold increase in risk for psychiatric illness, including psychosis (Prasad et al., 2015). In the studies outlined above, craniofacial dysmorphology was quantified in terms of overall differences in craniofacial shape between cases and controls. These analyses, involving generalised Procrustes registration in order to describe the variation of individual shapes around a mean (Goodall, 1991), have focussed conventionally on all types of deformations at given locations on surfaces, including those that operate uniformly across the surface at large scale. However, as recently described (Katina, 2012), it is now possible to resolve more complex changes in *non-affine space* in which deformation at given locations is not assumed to be uniform, to reflect the practical reality that each location often has a distinct structural environment (Wen et al., 2012; Hufnagel, 2015).

Such changes may considerably extend the incisiveness of geometric morphometrics for resolving craniofacial dysmorphology in greater topographical detail and hence enhance biological interpretation of craniofacial dysmorphology as an index of brain dysmorphogenesis. Therefore, given the enduring controversy regarding an early developmental basis to bipolar disorder, we here report an initial study of craniofacial dysmorphology in this illness in *non-affine space*.

## 2 Methods

### 2.1 Participants

Approval for this study was obtained from the Research Ethics Committee of St. John of God Hospital, Stillorgan, Co. Dublin, in accordance with the Declaration of Helsinki. All subjects were adults between the ages of 18–65 who gave written, informed consent to their participation following a complete description of the study.

Patients with bipolar disorder were recruited among those having this clinical diagnosis on admission to St. John of God Hospital, a general adult psychiatry hospital in Dublin, Ireland, or treated as outpatients by the Cluain Mhuire Centre, an outpatient mental health service in Dublin associated with St. John of God Hospital. A diagnosis of bipolar I disorder was established using the Structured Clinical Interview for DSM-IV. Participants included patients with either a first manic episode or a recent manic relapse of bipolar disorder independent of previous polarity or psychotic features. Additionally, we recruited schizophrenia patients with either a first psychotic episode or a recent psychotic relapse using similar procedures.

Control subjects were recruited from the administrative, clinical and ancillary staff of St John of God Hospital, Cluain Mhuire Centre and associated community facilities. Each prospective control was interviewed and excluded if they had a personal or family history of major affective disorder, psychosis or suicide in a first-degree relative according to Family History Research Diagnostic Criteria (Baker et al., 1987).

To exclude ethnic differences in craniofacies, all subjects, their parents and grandparents originated from and were born on the island of Ireland [Republic of Ireland or Northern Ireland], Scotland, Wales or England; all were white. Subjects were questioned about any craniofacial trauma or surgery and individuals who reported such events or evidenced beards or moustaches were excluded. Patients, together with control subjects, were recruited and assessed in an identical manner, in close temporal contiguity, by the same investigators over the course of a common study protocol. There were 21 patients with bipolar disorder [13 males, 8 females; mean age 35.8 (standard deviation (SD) 10.8), range 20–59 years] and 45 control subjects [18 males, 27 females; mean age 33.3 (SD 11.3), range 18–64 years]. Twenty-one patients with schizophrenia [17 males, 4 females; mean age 32.6 (SD 12.3), range 18–62 years] were also studied.

### 2.2 3D Laser surface imaging and image processing

**Fig. 1:**
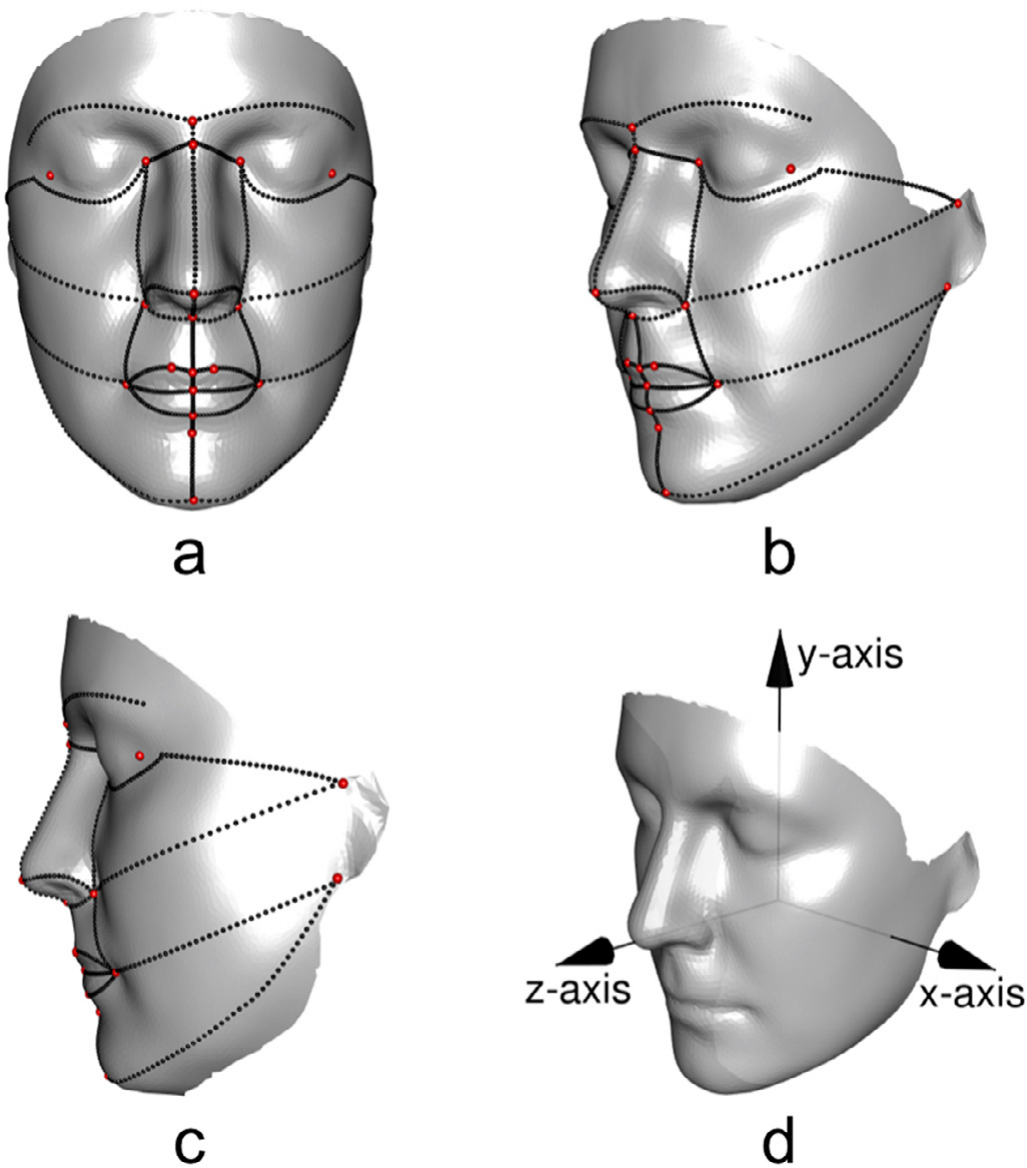
Anatomical landmarks (red) and semi-landmarks on anatomical and intermediate curves (black) on facial surfaces in (a) coronal, (b) coronal-sagittal oblique and (c) sagittal views, with (d) *x, y* and *z* axes used in Figs. 2 and 3 superimposed on coronal-sagittal oblique view. (For interpretation of the references to color in this figure legend, the reader is referred to the web version of this article.)

Facial surfaces were recorded by a single investigator (BK), using a portable, hand-held 3D laser imaging system (Polhemus FastScan, Vermont, USA), as described previously (Hennessy et al., 2007, 2010; Prasad et al., 2015). Typical surfaces, consisting of ~80,000 points [~160,000 triangles] (Fig. 1), are as shown previously in detail (Hennessy et al., 2007; Prasad et al., 2015). Incomplete data due to hair and complex folded surfaces were resolved using a fully automatic algorithm (Hu et al., 2012), which we have shown to achieve an optimal balance between performance and triangle manipulation (Rojas et al., 2014).

### 2.3 Facial landmarks

Following preprocessing, craniofacial shape was characterised first by locating manually 23 biologically homologous anatomical landmarks [nine on the midline and 14 as right and left counterparts of each of seven lateralised points (Prasad et al., 2015); these landmarks, shown in Fig. 1, were identified by a single investigator (SK), who was unaware of patient-control status.

This landmark set was augmented by 725 geometrically homologous semi-landmarks [also known as pseudo- or interpolated landmarks] on anatomical and intermediate curves (ridges, valleys, geodesic; Fig. 1) to improve description of the face in regions where anatomical landmarks are not present (Katina et al., 2016). If semi-landmarks were missing on both the right and left sides of the face, these were estimated by thin-plate spline (TPS) warping (see Dryden and Mardia, 2016) of a symmetric facial template onto a given facial surface using the anatomical landmarks and semi-landmarks as anchoring points (Gunz et al., 2008; Senck et al., 2015); if semi-landmarks were missing only on one side of the face, the opposite side of the face was warped to the side of interest. The positions of the semi-landmarks on each face were adjusted iteratively by sliding to create points that are geometrically homologous with respect to the template; this was achieved by minimising bending energy between the template and each facial shape, which has the effect of removing artificial deformation (Katina et al., 2016).

This sliding technique was applied together with generalized Procrustes analysis (GPA; Dryden and Mardia, 1998) to match the entire set of faces by minimising the Procrustes shape distance across location, orientation, and scale. This also allows Procrustes mean shape to be computed, which was used as a template for a second stage of iterative adjustment in order to improve accuracy. These processes were repeated until convergence. For subsequent analysis, Procrustes shape co-ordinates (PSC) were used.

### 2.4 Geometric morphometrics and visualisation

The nature of variation in samples of shapes is commonly explored through principal component analysis (PCA), based on the covariance matrix of the PSC (Dryden and Mardia, 2016); this represents the majority of the variation in the data through a smaller number of new variables that are constructed as linear combinations of the original variables (Prasad et al., 2015). As applied to 2D shapes (Bookstein, 1989), the concept of bending energy, which measures shape change by analogy with the physical process of surface deformation, was implemented. This specified that PCA was applied to resolve the more complex changes (non-linear, non-uniform) in *non-affine space*.

In all instances, PCA was applied to resolve and visualise differences between (a) bipolar patients and controls and (b) schizophrenia patients and controls, with adjustment for age and sex by a linear regression model in principal component (PC) scores (Prasad et al., 2015). These geometric morphometric methods and statistical analyses were implemented by direct coding in the R statistical computing environment (Core Team, 2020). Further details on these methods, and code to implement them, are available from the authors on request. For statistical tests, the significance level α was set at 0.05 and the results presented in terms of *t*-statistics [*t*, using 82 degrees of freedom calculated as the number of patients and control subjects (87) minus the number of parameters in the regression model (5, i.e. PC scores, age, sex, diagnosis and sex × diagnosis interaction)] and conventional *p*-values (*p*); Bonferroni adjustments were applied to control the family-wise (generalized Type I) error rate in a conservative manner for comparison of each patient group across the five PCs examined.

## 3 Results

### 3.1 Geometric morphometrics

PCA in *non-affine space* identified PCs 1–5 as explaining 51.0% of variance in facial morphology (Table 1; in this table only five PCs, i.e. those each accounting for > 5% of variance, are included). A regression model with terms for age and sex was adopted to ensure that these possible effects were incorporated. PC1 and PC3 varied with age, while PC2 and PC3 distinguished the sexes (each *p* < 0.01; Bonferroni adjustment was not used as age and sex were included in regression models regardless of significance). These relationships are in accordance with a long-standing and extensive literature on sex- and age-related variations in normal human craniofacial shape by ourselves and others (Hennessy et al., 2002; Ilankovan, 2014; Kersterke et al., 2016) and thus are not considered further. Following adjustment for age and sex, PC1 distinguished bipolar disorder cases from controls (*p* = 0.003, Table 1; Bonferroni significance level 0.05/10 = 0.005), with no diagnosis × sex interaction; no PC was informative in distinguishing schizophrenia cases from controls at the indicated level of significance. Following primary analyses in *non-affine space*, secondary analyses in *overall shape space* and *affine space* were conducted and were not informative (see Supplementary material Tables S1 and S2).

**Table 1:** Principal component analysis for *non-affine space.*

	Variance		Bipolar *vs* controls		Schizophrenia *vs* controls	
PC	Explained%	Cumulative%	*t*	*p*	*t*	*P*
PC1	18.0%	18.0%	3.015	0.003	–1.055	0.295
PC2	11.2%	29.2%	–0.075	0.940	0.255	0.800
PC3	8.4%	37.6%	–0.898	0.372	–0.282	0.779
PC4	7.0%	44.7%	1.719	0.090	–0.192	0.848
PC5	6.3%	51.0%	–0.937	0.352	0.507	0.614

Variance and cumulative variance explained by each principal component (PC), with probability values adjusted for age and sex by a linear regression model for each PC in distinguishing bipolar and schizophrenia patients from controls; *p* values should be compared to the Bonferroni adjusted significance level 0.05/10 = 0.005.

### 3.2 Visualisation

**Fig. 2:**
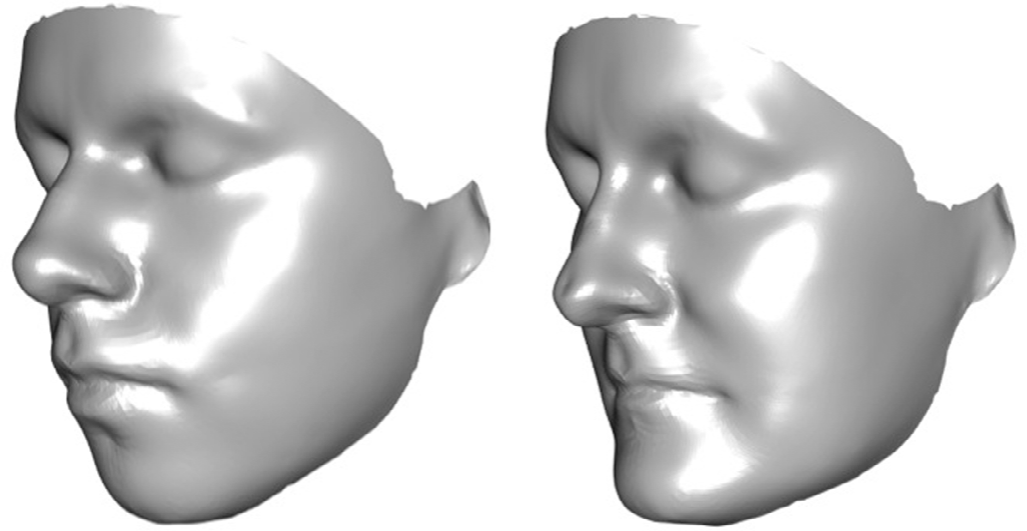
Visualization of PC1 of *non-affine space* as plain surfaces on coronal-sagittal oblique view for (left) control shape and (right) patient shape (each magnified × 3).

**Fig. 3:**
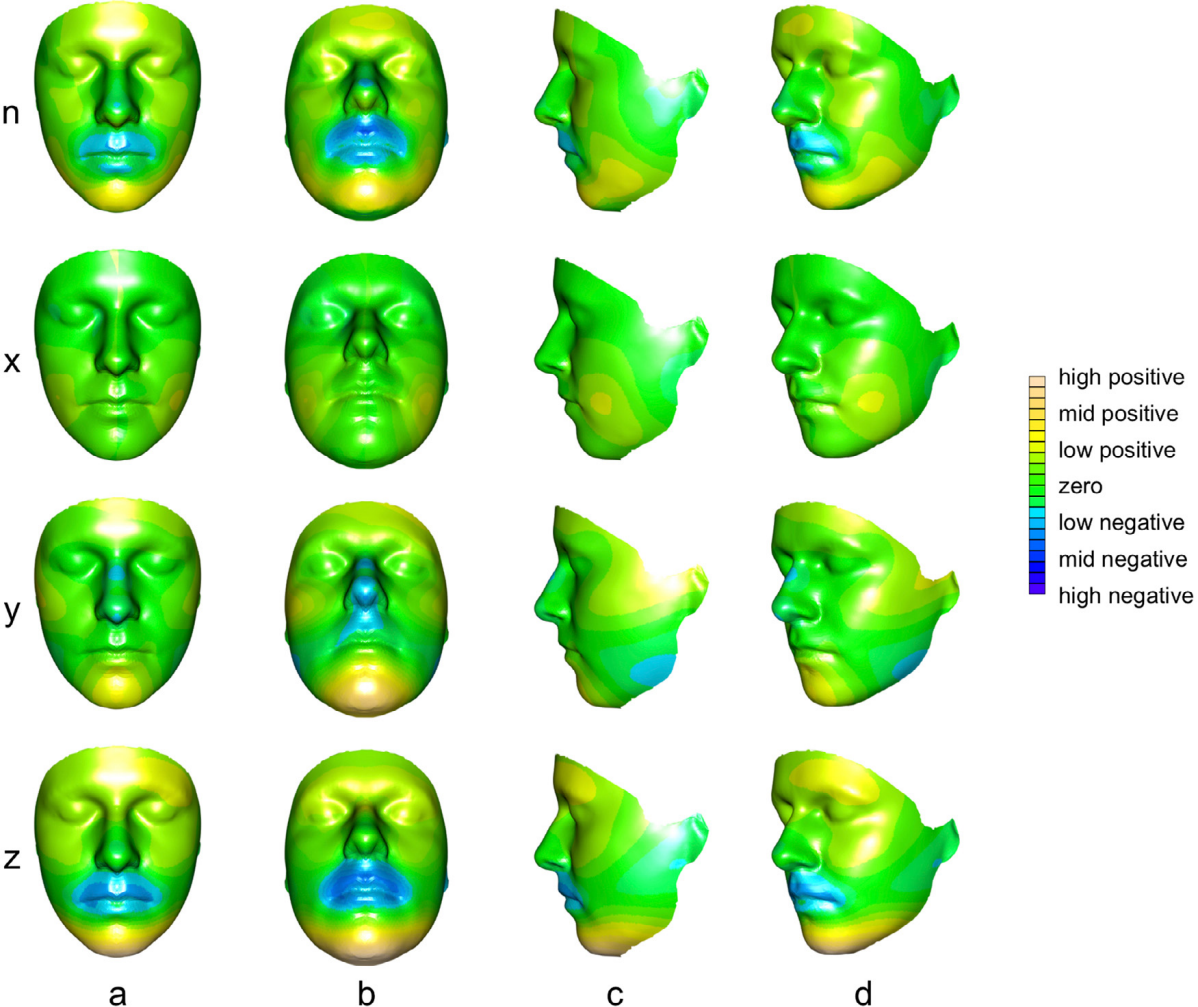
Visualisation of PC1 of *non-affine space* as control mean with colour-coded directional differences from control mean to bipolar mean (all magnified × 3). Euclidean distances: n, Euclidean distance in normal direction, i.e. along perpendicular direction from each point on the facial surface; *x*, distance in *x*-axis direction from each point on the facial surface; *y*, distance in *y*-axis direction from each point on the facial surface; *z*, distance in *z*-axis direction from each point on the facial surface. Views: (a) coronal; (b) transverse-coronal 22.5°; (c) sagittal; (d) coronal-sagittal oblique; left side of subject shown on right side of image, in accordance with radiological convention. Inset: colour scale for 3D distances where positive [from mid-green, through yellow to brown] indicates values for bipolar cases > controls and negative [from mid-green, through blue to purple] indicates values for bipolar cases < controls. (For interpretation of the references to color in this figure legend, the reader is referred to the web version of this article.)

These findings for bipolar disorder in *non-affine space* were given biological import through visualisations of PC1 as bipolar-control differences in facial shape. The magnitude of the absolute difference between control and case means is relatively small and the corresponding shape change is subtle. In order to visualise the nature of these differences more effectively, the displayed images magnify the difference between cases and controls by a factor of three in each direction. More specifically, if the control and case means on the PC scale are represented as z_1_ and z_2_ respectively, where z_1_ has the smaller value, then the displayed control shape lies at *z_1_* – 3(*z_2_* – *z_1_*) and the displayed case shape at *z_2_* + 3(*z_2_* – *z_1_*). Plain surfaces that correspond to magnified control shape and case shape, after adjusting for age and sex, are shown in Fig. 2. More quantitatively, data are shown as Euclidean distances from control shape to bipolar shape at each point on the facial surface for the normal [n] direction, i.e. perpendicular to the local surface area, and for orthogonal [*x, y, z*] components, after adjusting for age and sex (Fig. 3).

Using terminology for phenotypic variations that includes craniofacial topographies from *Elements of Morphology* (Allanson et al., 2009; Prasad et al., 2015) these visualisations indicate the following ectodermally derived surface features of (i) head and face, (ii) periorbital region, (iii) nose and philtrum, and (iv) lips, mouth and oral region to statistically distinguish bipolar cases from controls:

#### 3.2.1 Face

Upper face: slight (i.e. lower ranges of colour scales in either direction from zero) prominence of forehead and supraorbital ridges (Fig. 3, row n, columns a and b; row *z*, columns a–d).

Maxilla and midface: slight prominence of cheeks (Fig. 3, row n, columns a, b and d; row *z*, columns a and d); slight retrusion, narrowing and elongation of upper posterior midface (Fig. 3, row n, column c; rows *x* and *y*, column c; row *z*, column c); slight widening of lower midface (Fig. 3, row *x*, columns a–d).

Premaxilla: marked (i.e. upper ranges of colour scales in either direction from zero) retrusion and shortening of premaxilla (Fig. 3, row n, columns a-d; row *y*, column b; row *z*, columns a–d).

Mandible and chin: marked prominence and elongation of anterior jaw and chin (Fig. 3, row n, columns a–d; row *y*, columns a and b; row *z*, columns a–d); slight shortening of the posterior jaw (Fig. 3, row *y*, columns c and d).

Each of the above dysmorphologies appeared symmetrical, with the exception of slight prominence of the forehead, which appeared more evident on the left side (right side of images, in accordance with radiological convention).

#### 3.2.2 Periorbital region

Slight prominence of the eyes (Fig. 3, row n, columns a and b; row *z*, columns a–d). This dysmorphology appeared symmetrical.

#### 3.2.3 Nose and philtrum

Marked retrusion and shortening of nasal tip, nostrils, nasal base and philtrum, with narrowing of nasal ridge and philtrum (Fig. 3, row n, columns a–d; row *y*, columns a–d; row *z*, columns a–d). These dysmorphologies appeared symmetrical.

#### 3.2.4 Lips, mouth and oral region

Marked retrusion of upper and lower lips and mouth, with narrowing of upper lip and mouth (Fig. 3, row n, columns a–d; row *y*, columns a–d; row *z*, columns a–d). These dysmorphologies appeared symmetrical.

## 4 Discussion

In this study we report the 3D topography of craniofacial dysmorphology in bipolar disorder in *non-affine space*. This analysis reveals complex deformations that can be related to the known developmental biology of the human face and its relationship to brain morphogenesis, so as to provide information on putative mechanisms of brain dysmorphogenesis in this disorder. It should be emphasised that the term ‘deformations’ is used in its technical sense: these findings are too subtle to be noted qualitatively on visual inspection of any individual patient, and are evident only on quantitative assessment of patient groups using 3D imaging technology and analysis by geometric morphometrics. On a background of minor dysmorphologies of the upper face, maxilla, midface and periorbital region, the main features in bipolar disorder are (a) retrusion and shortening of the premaxilla, nose, philtrum, lips and mouth (the frontonasal prominences), with (b) protrusion and widening of the mandible-chin.

Over early fetal life, the brain and face share a common embryological origin during which tissues from ectodermally derived primordia interact intimately in terms of molecular signalling and physical influences; thus, disruption to processes regulating early brain development are accompanied by facial anomalies (DeMyer et al., 1964; Marcucio et al., 2015). Five primordia give rise to five developmental fields that ultimately fuse over early fetal life to create facial form: the frontonasal process, which enjoys the most intimate relationship with development of the forebrain; paired maxillary processes; paired mandibular processes that relate less intimately to brain development (Schneider et al., 2001; Marcucio et al., 2015).

Critically, the topography of dysmorphology in bipolar disorder implicates impairment of early development in the frontonasal process that would predict cerebral dysmorphogenesis, particularly in the forebrain; recent studies and meta-analyses in bipolar disorder have indeed reported reductions in frontal grey matter volume (Birur et al., 2017; Chang et al., 2018), reductions in frontal white matter connectivity with more posterior regions (Birur et al., 2017), and reductions in functional integrity in frontoparietal and cingulo-opercular networks that correlate with extent of cognitive impairment (Sheffield et al., 2017). This impairment of early development in the frontonasal process was accompanied by the prominence of early development in the mandibular processes. This may reflect compensatory events, either embryologically to sustain overall facial morphology via an adjacent developmental field less closely related to brain development, or involving physical interactions between adjacent developmental fields.

Though our findings in bipolar disorder appeared robust, given previous findings of ourselves and others (Buckley et al., 2005; Hennessy et al., 2007, 2010; Prasad et al., 2015) the marginality of findings in schizophrenia was unexpected. This may reflect, at least in part, the present paucity of female schizophrenia cases relative to female controls and these few female schizophrenia cases being among the older subjects ascertained, as such variations can distort geometric morphometric analyses by increasing age-related confounding effect on shape. Notably, other investigators using conservative diagnostic criteria, such as were applied here, have remarked on the increasing paucity of female SZ cases in research studies (Lewine et al., 1984; Iacono and Beiser, 1992; Longenecker et al., 2010). Confining analyses to males did not provide clarification; the findings were similar to those across both sexes.

Embryological data on the timeline of brain-face relationships over fetal life (Diewert and Lozanoff, 1993; Diewert et al., 1993) indicate that these evolve and approach postnatal morphology during gestational weeks 6 through weeks 19–20. On this basis, we have speculated (Hennessy et al., 2010) that primary dysmorphogenic events may take place during this period. The present findings refine these notions by emphasising frontonasal dysmorphology in bipolar disorder, whether due to genetic or environmental factors or involving gene-environment interactions. Frontonasal dysmorphology enjoys the most intimate relationship with development of the forebrain during the period of gestational weeks 9–10 through weeks 14–15 (Diewert and Lozanoff, 1993; Diewert et al., 1993). Indeed, using alternative embryological considerations, other authors have recently proposed similarly that abnormal development between the 10^th^ and 15^th^ week of gestation appears related to reduced brain volume in bipolar disorder (Vonk et al., 2014).

In the context of enduring controversies regarding the extent to which bipolar disorder does or does not have developmental origins (Sanches et al., 2008; Demjaha et al., 2012; Parellada et al., 2017), the present findings constitute ‘hard’, quantitative evidence for disruptive events operating over early fetal life. More specifically, these findings in *non-affine space* provide resolution of the topography of facial dysmorphology in bipolar disorder and, on embryological grounds, greater insight into brain dysmorphogenesis, including the putative timing of these events. Future studies should seek to disentangle the relative roles of early genetic and environmental adversities in the dysmorphogenic process(es) of bipolar disorder and the extent to which this may generalise across psychotic diagnoses.

## Funding

These studies were carried out under the auspices of the Face3D Consortium (www.face3d.ac.uk) funded by the Wellcome Trust [086901/Z/08/Z]. This agency had no further role in study design, collection, analysis and interpretation of data or in the decision to submit the manuscript for publication.

## Supplementary materials

Supplementary material associated with this article can be found, in the online version.

## Author statement

SK performed anatomical landmarking of images, geometric morphometric analysis and visualisation of imaging data; BDK and AL conducted ascertainment, clinical assessment and image acquisition of participants; MAR, FMS and PFW conducted primary processing of imaging data for geometric morphometric analysis; AMcD performed anatomical landmarking of images; RJH conducted training in image acquisition and curation and initial processing of imaging data; AWB performed geometric morphometric analysis and visualisation of imaging data; JLW coordinated the study, biological interpretation of findings, drafting and preparation of the final version.

All authors contributed to drafting and approved the final version of the manuscript.

## Declaration of Competing Interest

The authors report no conflict of interest.

## Appendix Supplementary materials

Image, application 1
