# Supplementary material for: Refining the resolution of craniofacial dysmorphology in bipolar disorder as an index of brain dysmorphogenesis
Source: Psychiatry Res. 2020 Sep;291:113243. doi: 10.1016/j.psychres.2020.113243 (PMC7487763; doi:10.1016/j.psychres.2020.113243)
Supplement: Supplementary file 1 [file mmc1.doc]

Supplementary material

Deformations at given (semi)landmark neighbourhoods on surfaces are analysed conventionally in *overall shape space*; this includes large scale deformations which operate in a linear manner across the whole object, referred to as *affine space*. However, as recently described (Katina, 2012), it is now possible to resolve more complex changes in *non-affine space* in which deformation at given locations is not assumed to be uniform, to reflect the practical reality that each location often has a distinct structural environment (Wen et al., 2012; Hufnagel, 2015); this is the focus and primary analysis in the present study (Table 1). Secondary analyses in *overall shape space* (Supplementary materials Table S1) and *affine space* (Supplementary materials Table S2), which were not informative, are provided below for completeness:

Table S1. Principal component analysis for *overall shape space*

|  | **Variance** | | **Bipolar *vs* controls** | | **Schizophrenia *vs* controls** | |
| --- | --- | --- | --- | --- | --- | --- |
| **PC** | **Explained**  **%** | **Cumulative**  **%** | ***t*** | ***p*** | ***t*** | ***p*** |
| **PC1** | 20.6% | 20.6% | 1.892 | 0.062 | –1.452 | 0.150 |
| **PC2** | 12.6% | 33.1% | –2.570 | 0.012 | –0.610 | 0.544 |
| **PC3** | 11.7% | 44.8% | 0.655 | 0.515 | –1.587 | 0.116 |
| **PC4** | 7.0% | 51.8% | 0.378 | 0.707 | –0.686 | 0.495 |
| **PC5** | 6.1% | 57.9% | –0.536 | 0.593 | –0.372 | 0.711 |

Variance and cumulative variance explained by each principal component (PC), with probability values adjusted for age and sex by a linear regression model for each PC in distinguishing bipolar and schizophrenia patients from controls; *p* values should be compared to the Bonferroni adjusted significance level 0.05/10 = 0.005.

Table S2. Principal component analysis for *affine space.*

|  | **Variance** | | **Bipolar *vs* controls** | | **Schizophrenia *vs* controls** | |
| --- | --- | --- | --- | --- | --- | --- |
| **PC** | **Explained**  **%** | **Cumulative**  **%** | ***t*** | ***p*** | ***t*** | ***p*** |
| **PC1** | 43.2% | 43.2% | 1.017 | 0.312 | –1.297 | 0.198 |
| **PC2** | 28.0% | 71.3% | 1.224 | 0.225 | 1.385 | 0.170 |
| **PC3** | 23.6% | 94.9% | –2.006 | 0.048 | 1.135 | 0.260 |
| **PC4** | 2.7% | 97.6% | 0.353 | 0.725 | 0.454 | 0.651 |
| **PC5** | 2.4% | 99.9% | 0.271 | 0.787 | –0.151 | 0.880 |

Variance and cumulative variance explained by each principal component (PC), with probability values adjusted for age and sex by a linear regression model for each PC in distinguishing bipolar and schizophrenia patients from controls; *p* values should be compared to the Bonferroni adjusted significance level 0.05/10 = 0.005.
